# Supplementary material for: New Polyene Macrolide Compounds from Mangrove-Derived Strain Streptomyces hiroshimensis GXIMD 06359: Isolation, Antifungal Activity, and Mechanism against Talaromyces marneffei
Source: Mar Drugs. 2024 Jan 8;22(1):38. doi: 10.3390/md22010038 (PMC10819995; doi:10.3390/md22010038)
Supplement: Supplementary file 1 [file marinedrugs-22-00038-s001.zip › marinedrugs-2797406-supplementary.pdf]

Supplementary Information

New Polyene Macrolide Compounds from Mangrove-Derived Strain *Streptomyces hiroshimensis* GXIMD 06359: Isolation, Antifungal Activity, and Mechanism against *Talaromyces marneffei*

Zhou Wang [1,2,†], Jianglin Yin [1,2,3,†], Meng Bai [1,2], Jie Yang [1,2], Cuiping Jiang [1,2], Xiangxi Yi [2], Yonghong Liu [1,2,*] and Chenghai Gao [1,2*]

[1] Institute of Marine Drugs, Guangxi University of Chinese Medicine, Nanning 530200, China; wangzhou2021@stu.gxtcmu.edu.cn (Z.W.); yinjianglin@126.com (J.Y.); xxbai2014@163.com (M.B.); jieyang202312@163.com (J.Y.); ping990120@foxmail.com (C.J.)

[2] Guangxi Key Laboratory of Marine Drugs, Guangxi University of Chinese Medicine, Nanning 530200, China; yixiangxi2017@163.com

[3] Guangxi Scientific Research Center of Traditional Chinese Medicine, Nanning 530200, China

* Correspondence: yonghongliu@scsio.ac.cn (Y.L.); gaoch@gxtcmu.edu.cn (C.G.)

† These authors contributed equally to this work.

List of Supporting Information

Figure S1. ^{1}H NMR (DMSO-d_6, 500 MHz) spectrum of **1**.

Figure S2. ^{13}C NMR (DMSO-d_6, 175 MHz) spectrum of **1**.

Figure S3. DEPT (DMSO-d_6, 175 MHz) spectrum of **1**.

Figure S4. HMQC spectrum of **1**.

Figure S5. HMBC spectrum of **1**.

Figure S6. COSY spectrum of **1**.

Figure S7. NOESY spectrum of **1**.

Figure S8. HR-ESI-MS spectrum of **1**.

Figure S9. Experimental UV spectrum of **1**.

Figure S10. ^{1}H NMR (DMSO-d_6, 500 MHz) spectrum of **2**.

Figure S11. ^{13}C NMR (DMSO-d_6, 175 MHz) spectrum of **2**.

Figure S12. DEPT (DMSO-d_6, 175 MHz) spectrum of **2**.

Figure S13. HMQC spectrum of **2**.

Figure S14. HMBC spectrum of **2**.

Figure S15. COSY spectrum of **2**.

Figure S16. NOESY spectrum of **2**.

Figure S17. HR-ESI-MS spectrum of **2**.

Figure S18. Experimental UV spectrum of **2**.

Figure S19. ^{1}H NMR (DMSO-d_6, 700 MHz) spectrum of **3**.

Figure S20. ^{13}C NMR (DMSO-d_6, 175 MHz) spectrum of **3**.

Figure S21. HMQC spectrum of **3**.

Figure S22. HMBC spectrum of **3**.

Figure S23. COSY spectrum of **3**.

Figure S24. NOESY spectrum of **3**.

Figure S25. HR-ESI-MS spectrum of **3**.

Figure S26. Experimental UV spectrum of **3**.

Figure S27. ^{1}H NMR (DMSO-d_6, 700 MHz) spectrum of **4**.

Figure S28. ^{13}C NMR (DMSO-d_6, 175MHz) spectrum of **4**.

Figure S29. DEPT (DMSO-d_6, 175 MHz) spectrum of **4**.

Figure S30. HMQC spectrum of **4**.

Figure S31. HMBC spectrum of **4**.

Figure S32. COSY spectrum of **4**.

Figure S33. NOESY spectrum of **4**.

Figure S34. HR-ESI-MS spectrum of **4**.

Figure S35. Experimental UV spectrum of **4**.

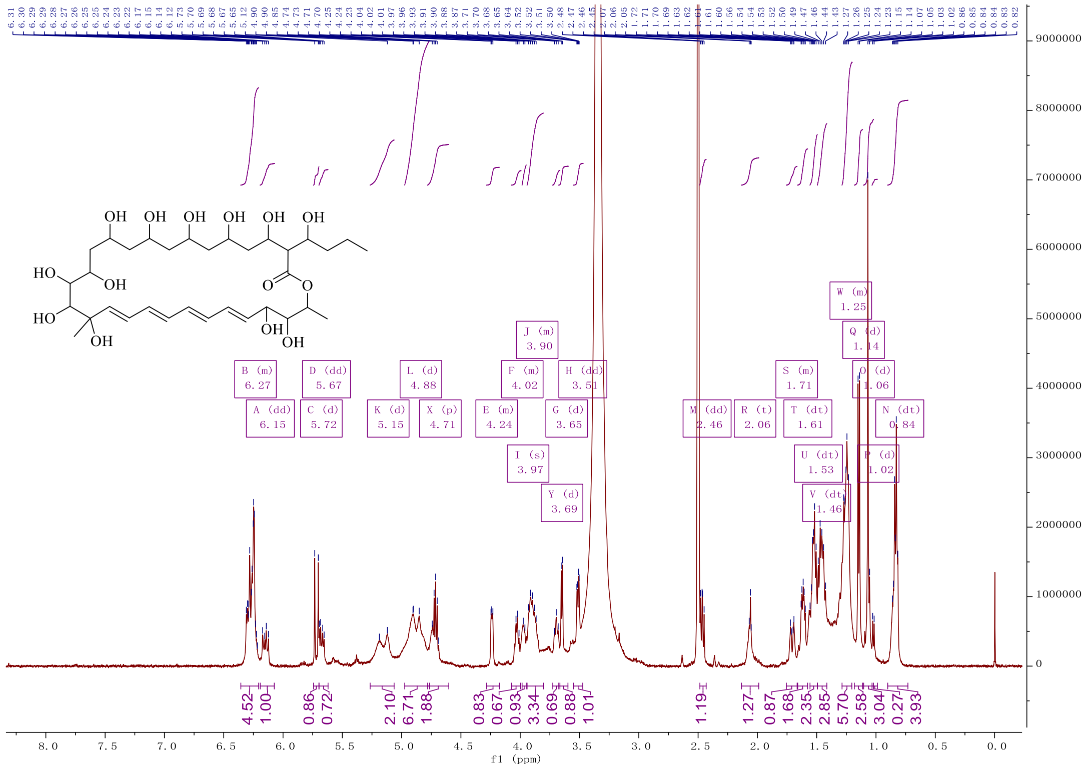

Figure S1. ^{1}H NMR (DMSO-d_6, 500 MHz) spectrum of **1**.

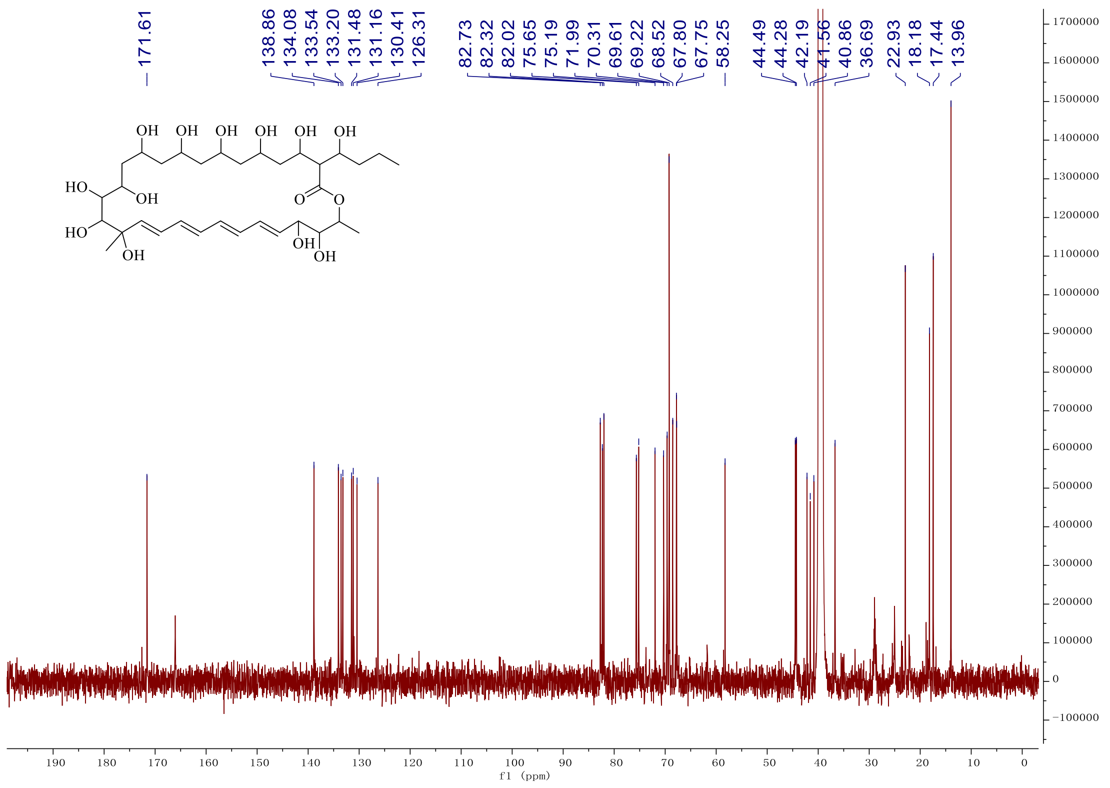

Figure S2. ^{13}C NMR (DMSO-d_6, 175 MHz) spectrum of **1**.

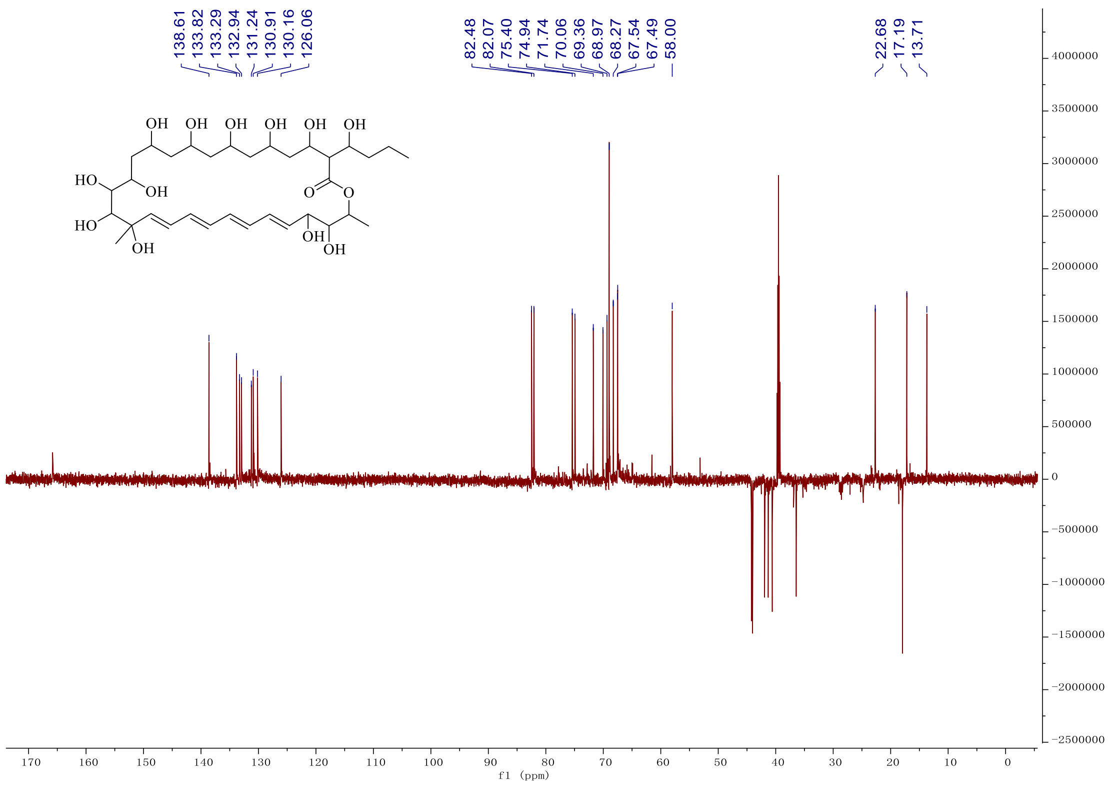

Figure S3. DEPT (DMSO-d_6, 175 MHz) spectrum of **1**.

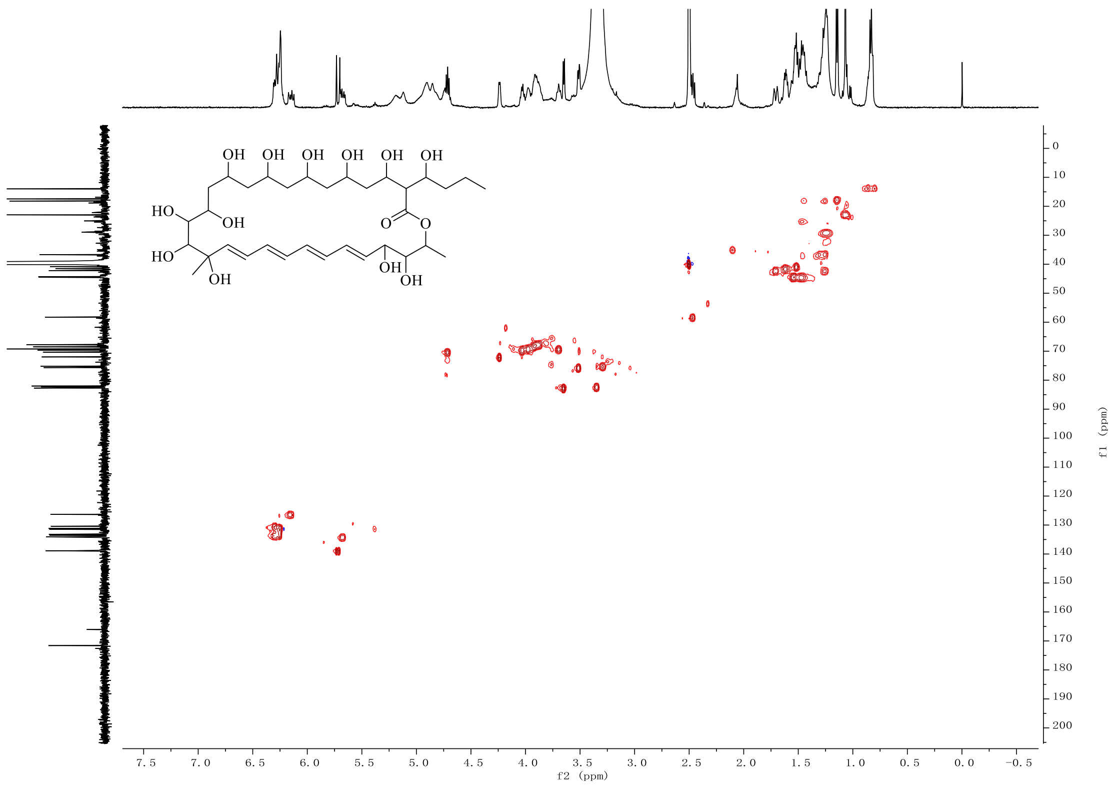

Figure S4. HMQC spectrum of **1**.

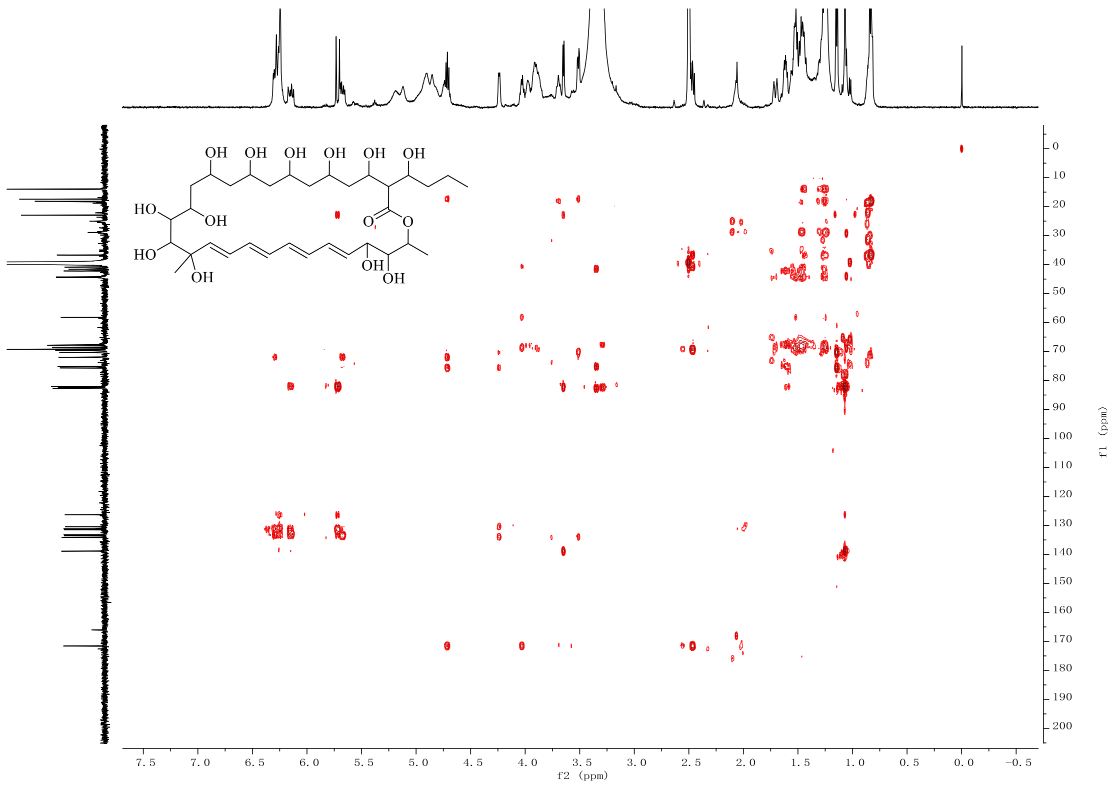

Figure S5. HMBC spectrum of **1**.

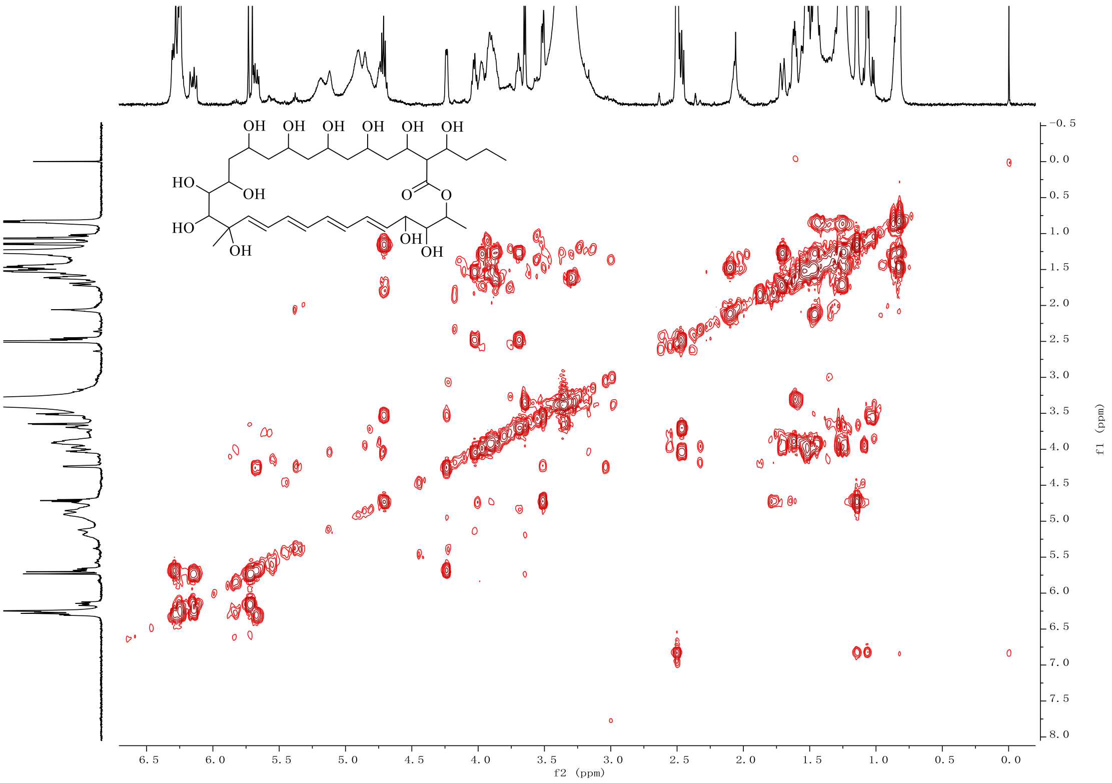

Figure S6. COSY spectrum of **1**.

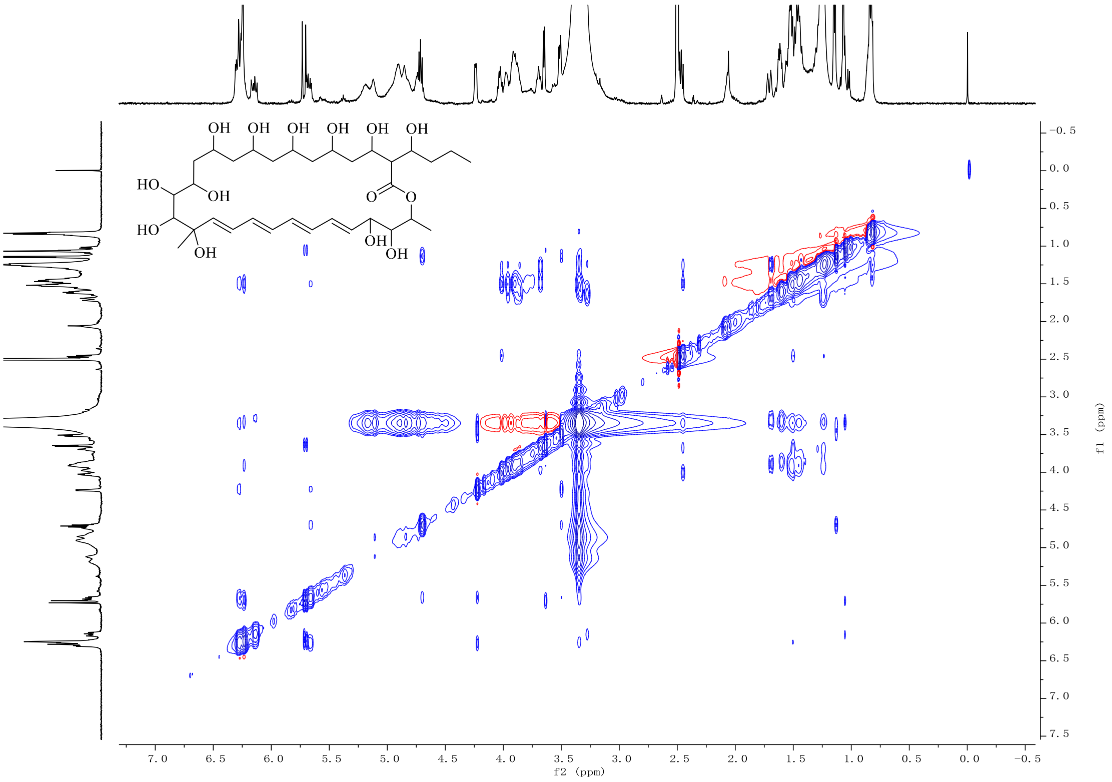

Figure S7. NOESY spectrum of **1**.

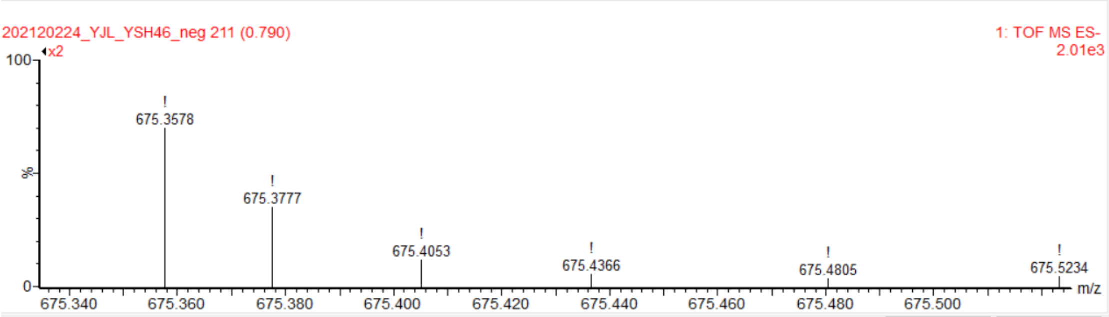

Figure S8. HR-ESI-MS spectrum of **1**.

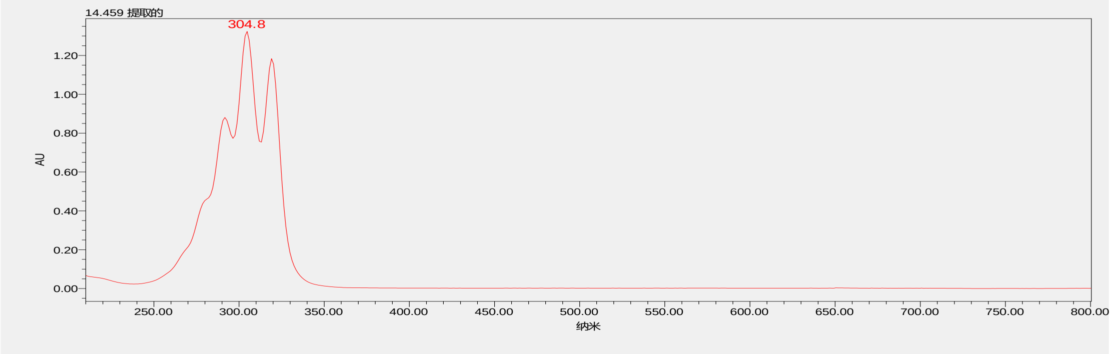

Figure S9. Experimental UV spectrum of **1**.

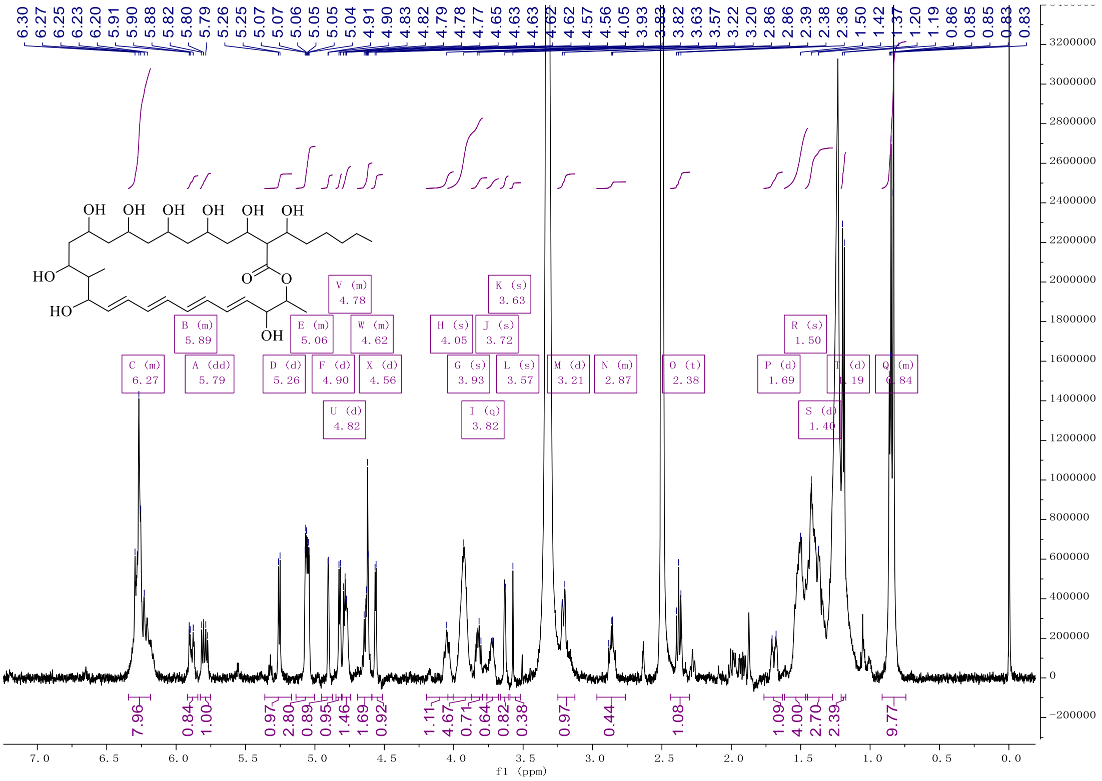

Figure S10. ^{1}H NMR (DMSO-d_6, 500 MHz) spectrum of **2**.

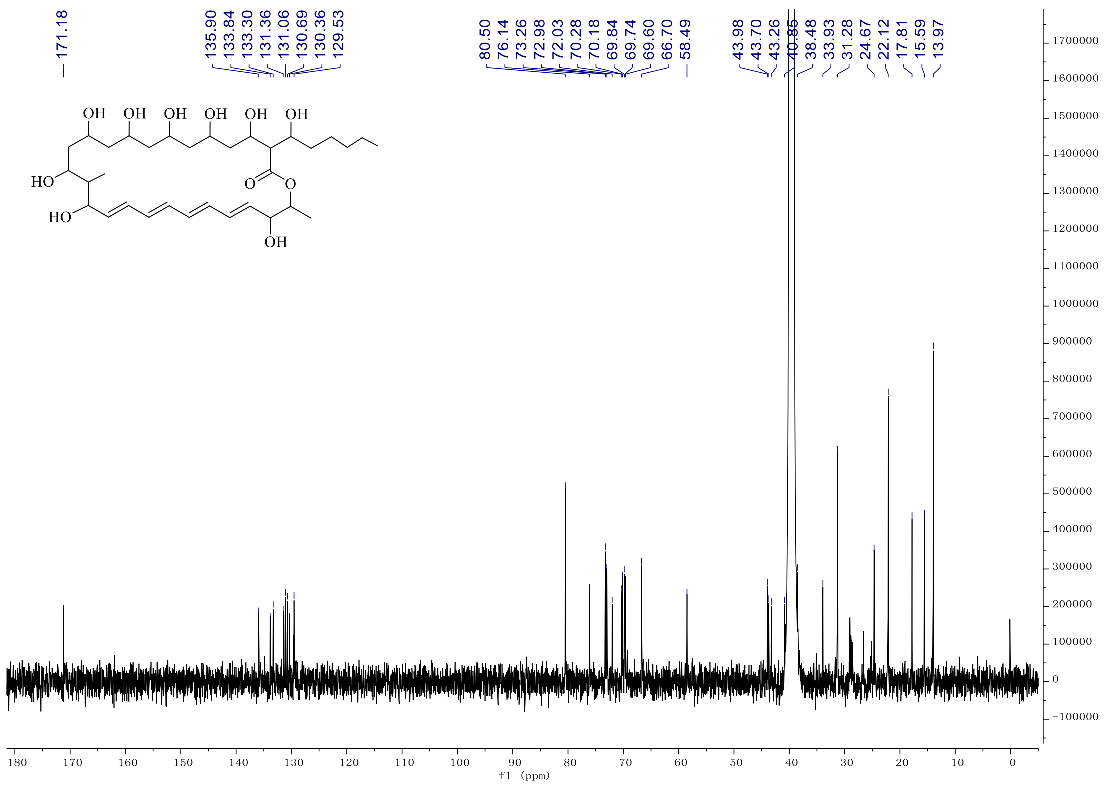

Figure S11. ^{13}C NMR (DMSO-d_6, 175 MHz) spectrum of **2**.

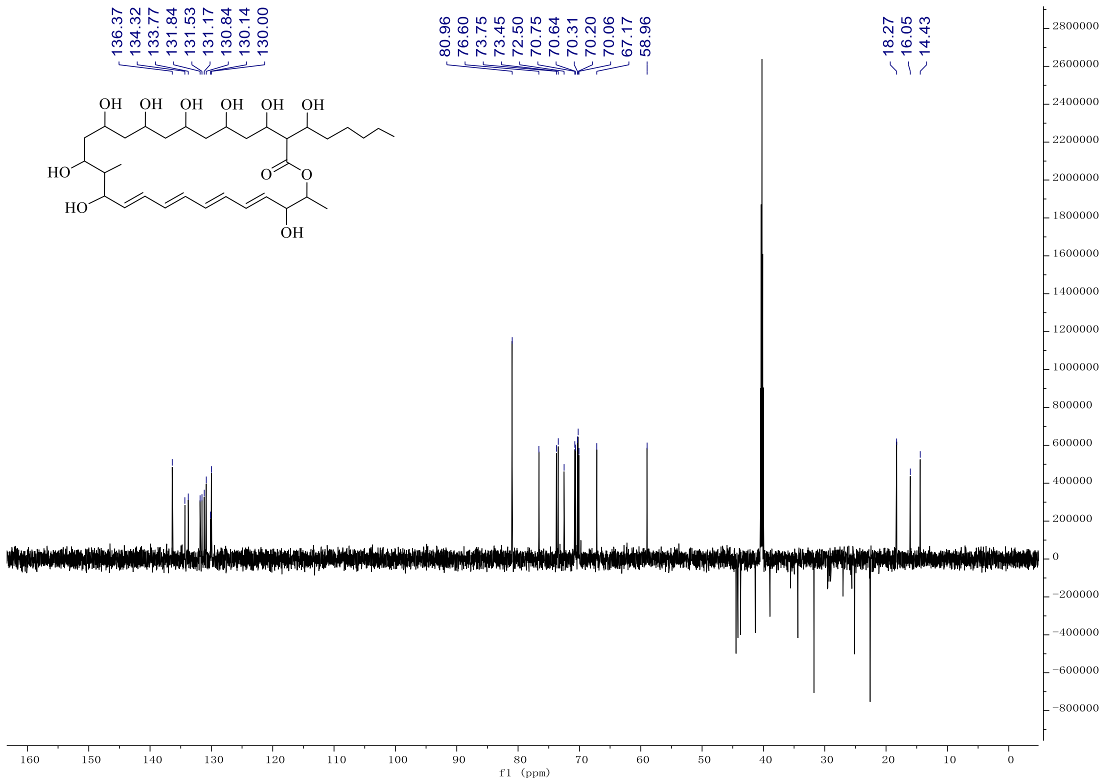

Figure S12. DEPT (DMSO-d_6, 175 MHz) spectrum of **2**.

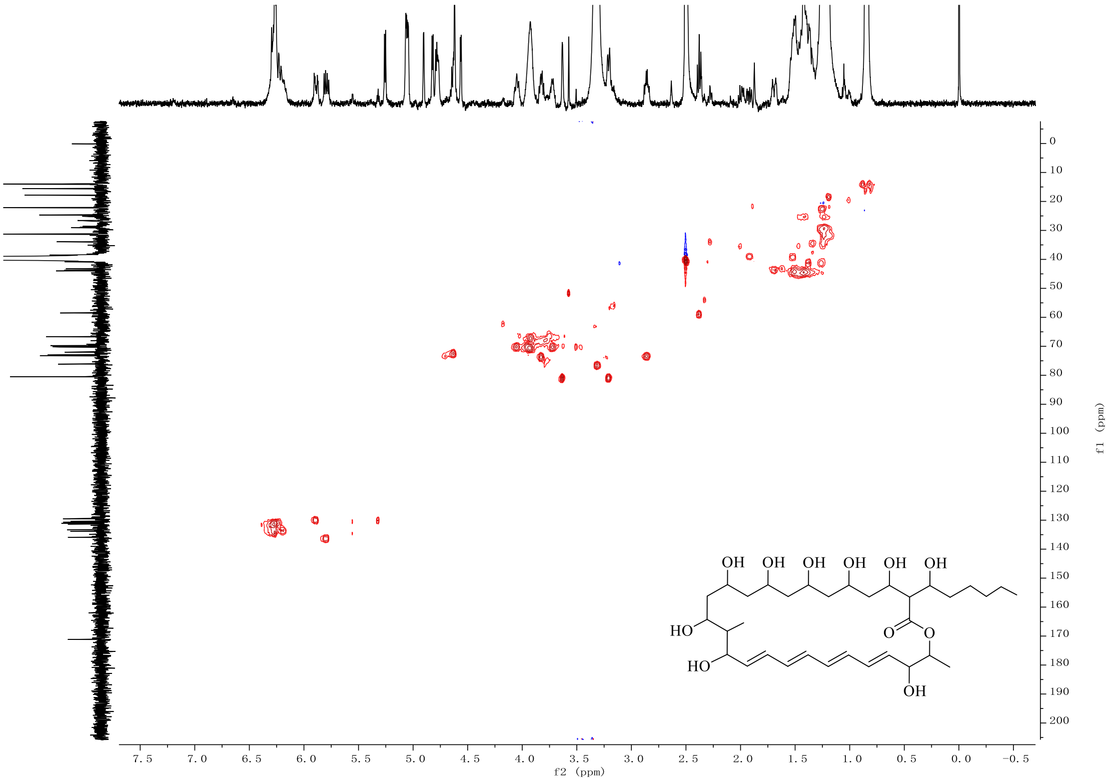

Figure S13. HMQC spectrum of **2**.

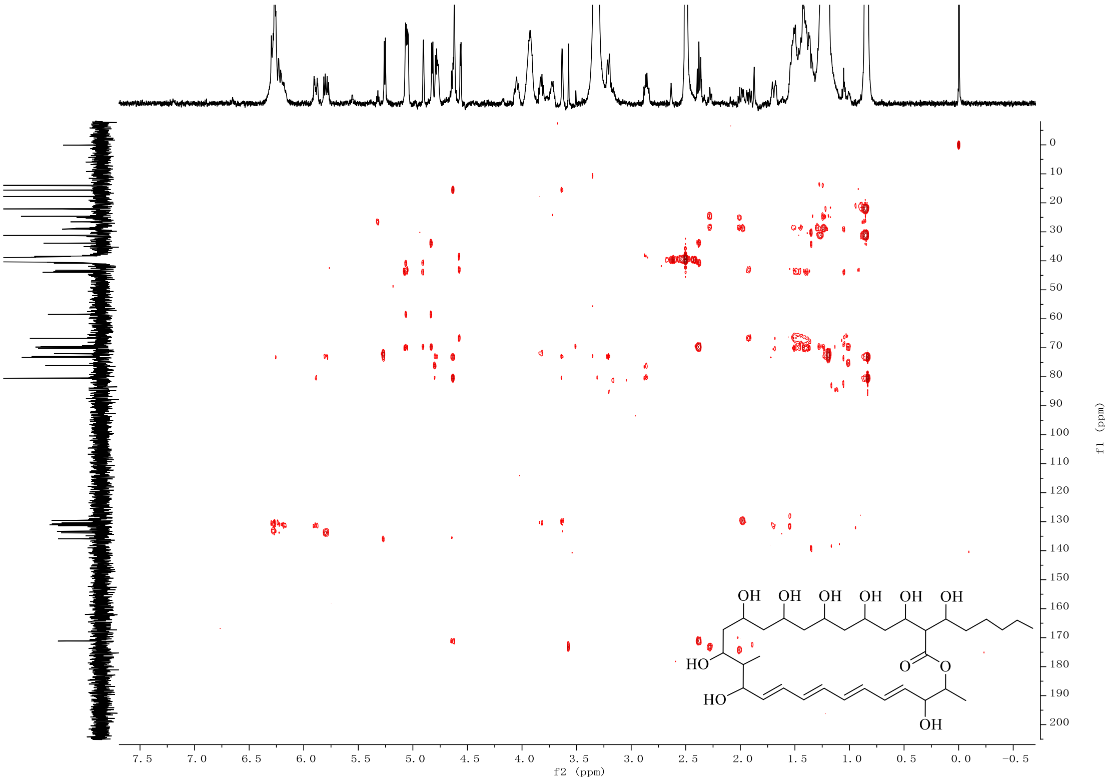

Figure S14. HMBC spectrum of **2**.

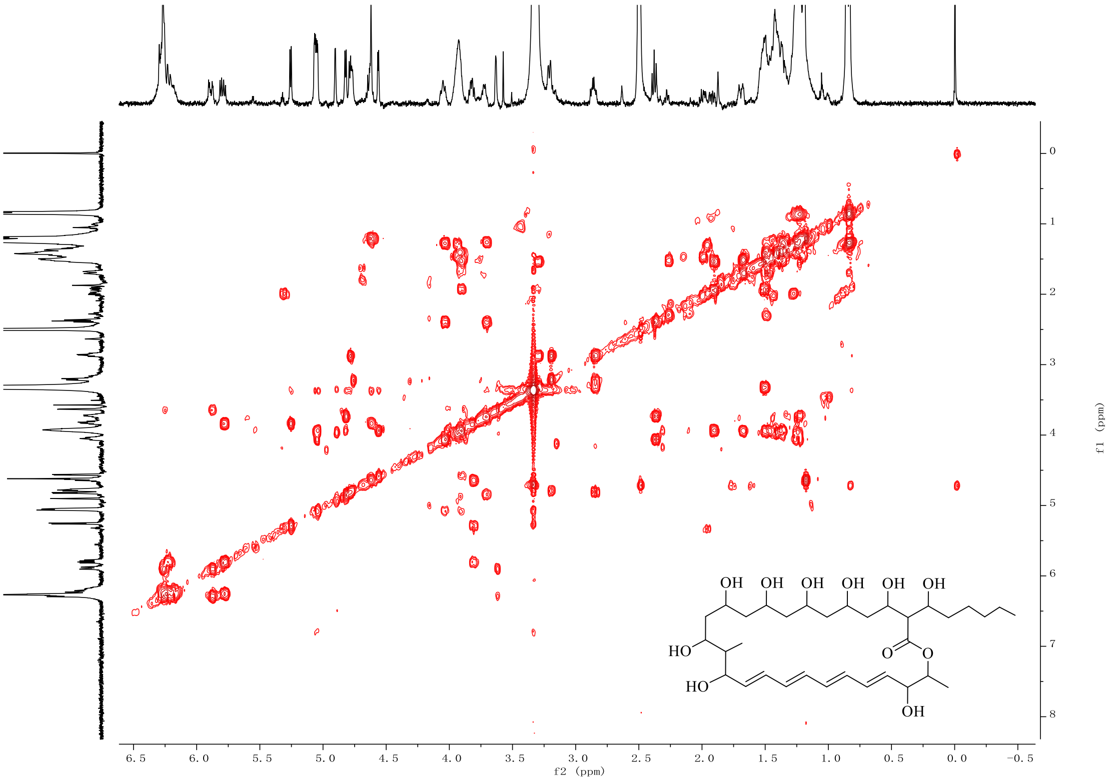

Figure S15. COSY spectrum of **2**.

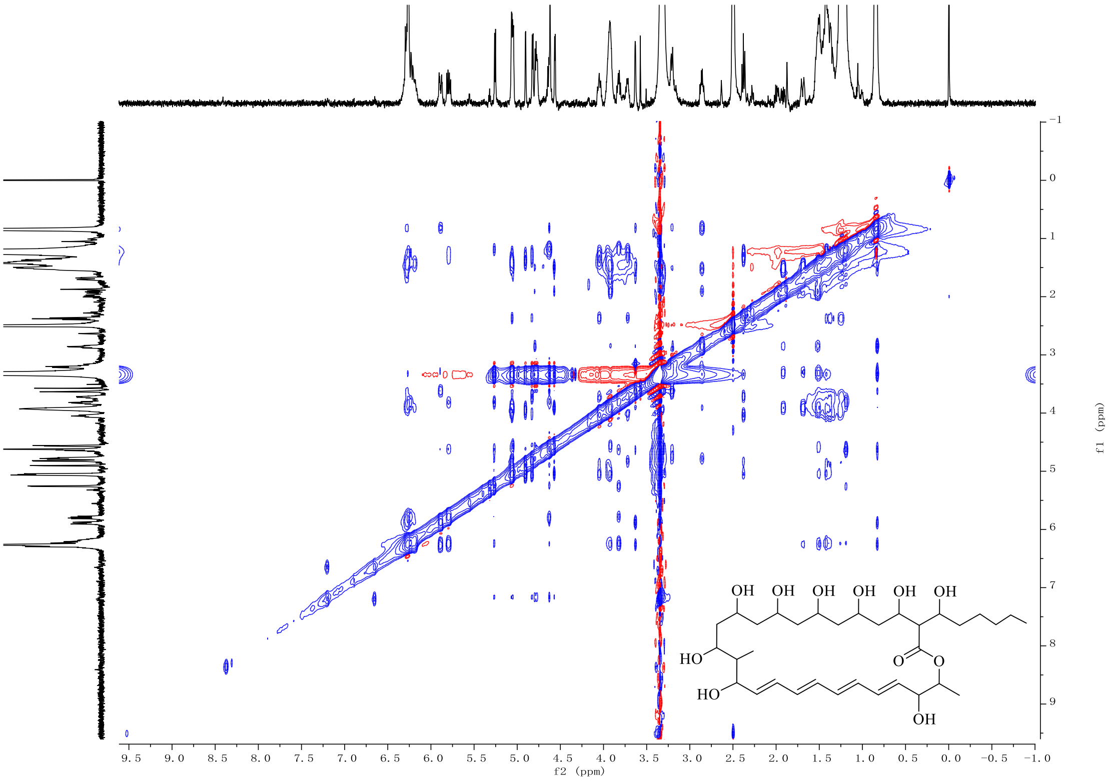

Figure S16. NOESY spectrum of **2**.

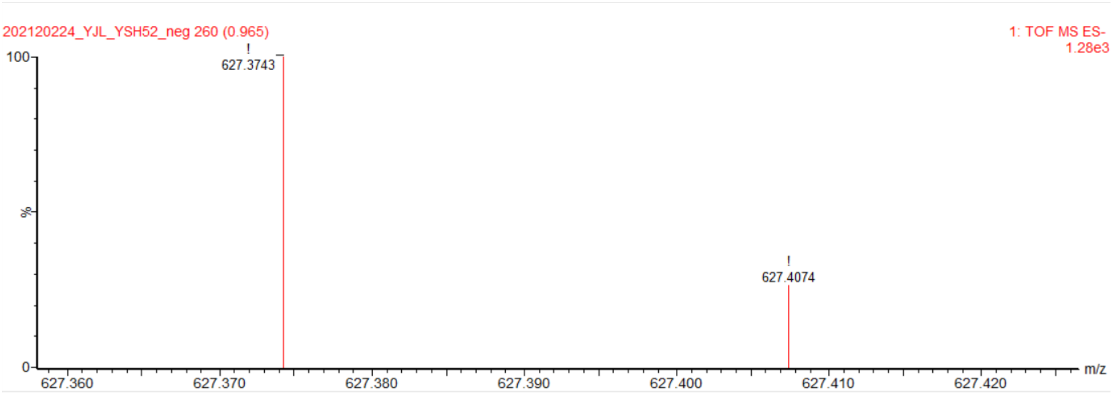

Figure S17. HR-ESI-MS spectrum of **2**.

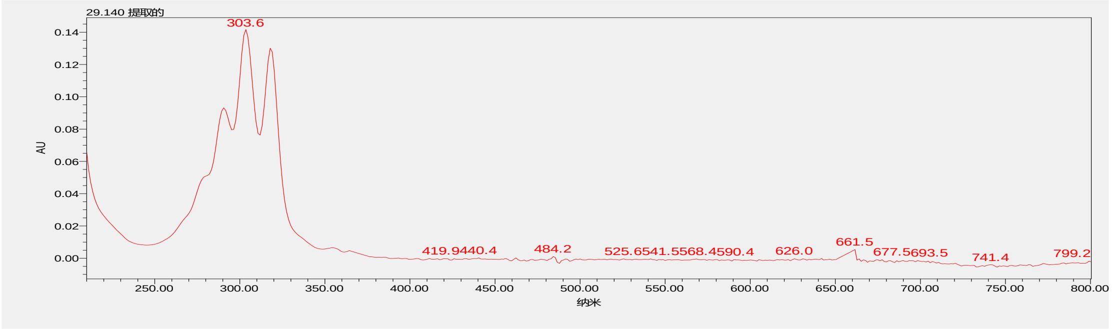

Figure S18. Experimental UV spectrum of **2**.

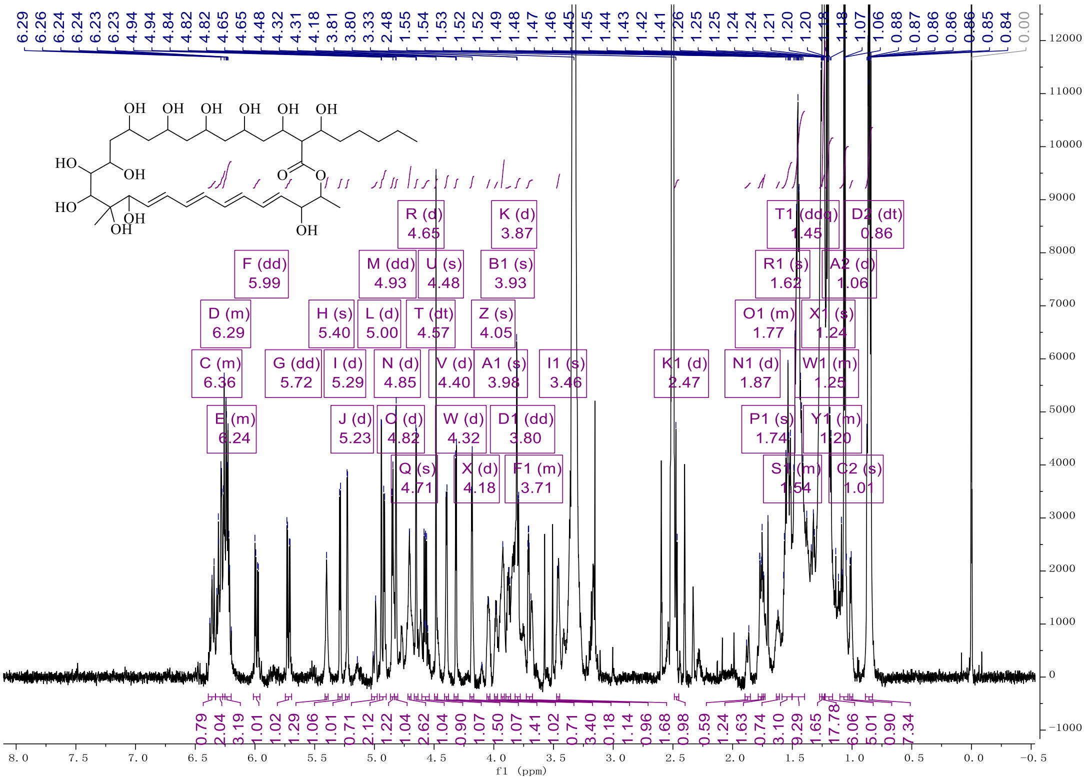

Figure S19. ^{1}H NMR (DMSO-d_6, 700 MHz) spectrum of **3**.

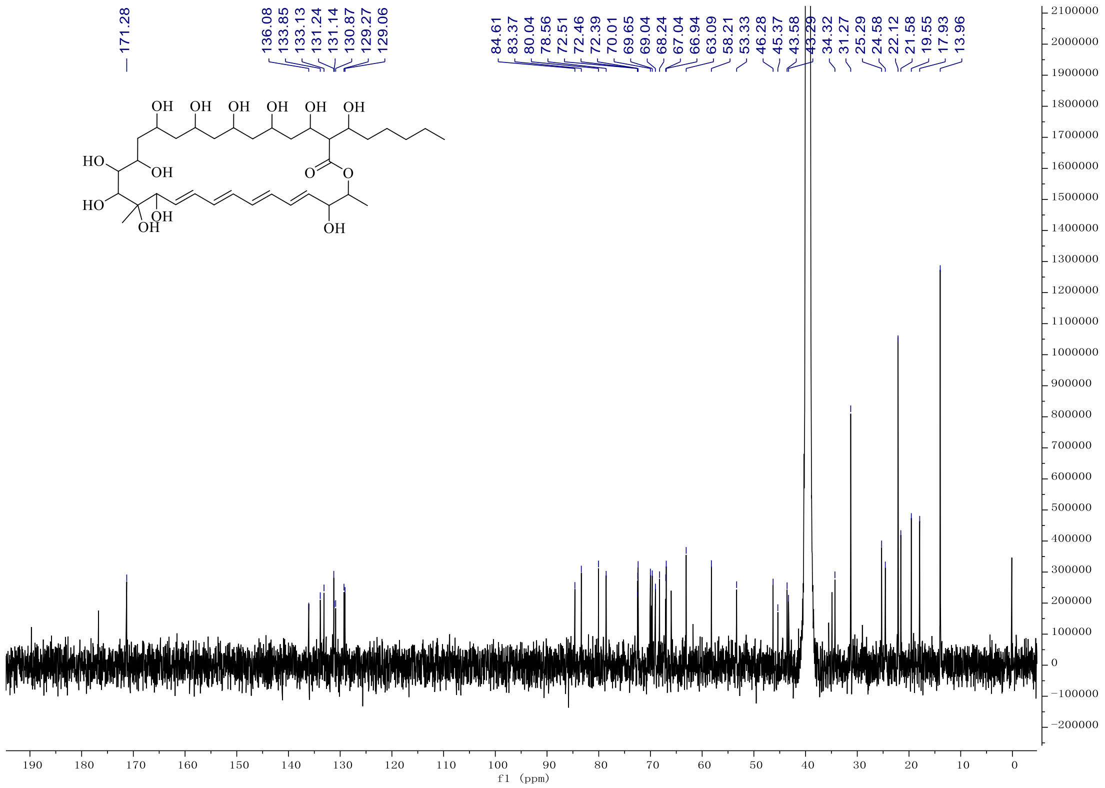

Figure S20. ^{13}C NMR (DMSO-d_6, 175 MHz) spectrum of **3**.

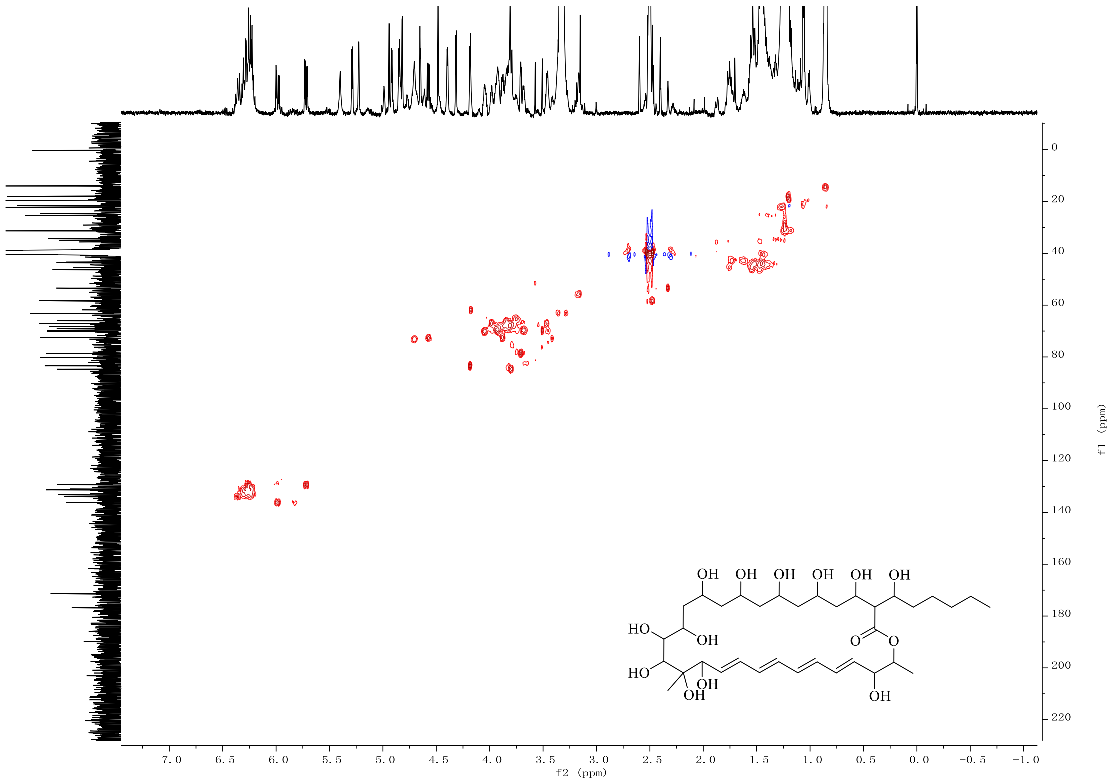

Figure S21. HMQC spectrum of **3**.

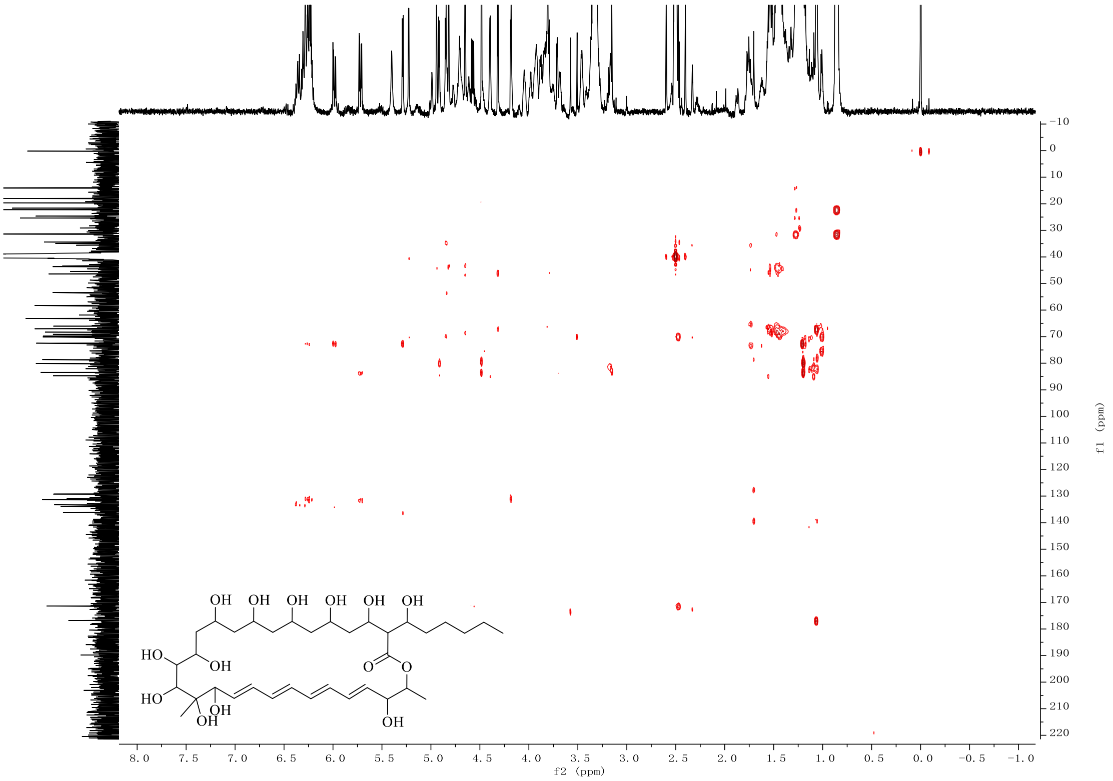

Figure S22. HMBC spectrum of **3**.

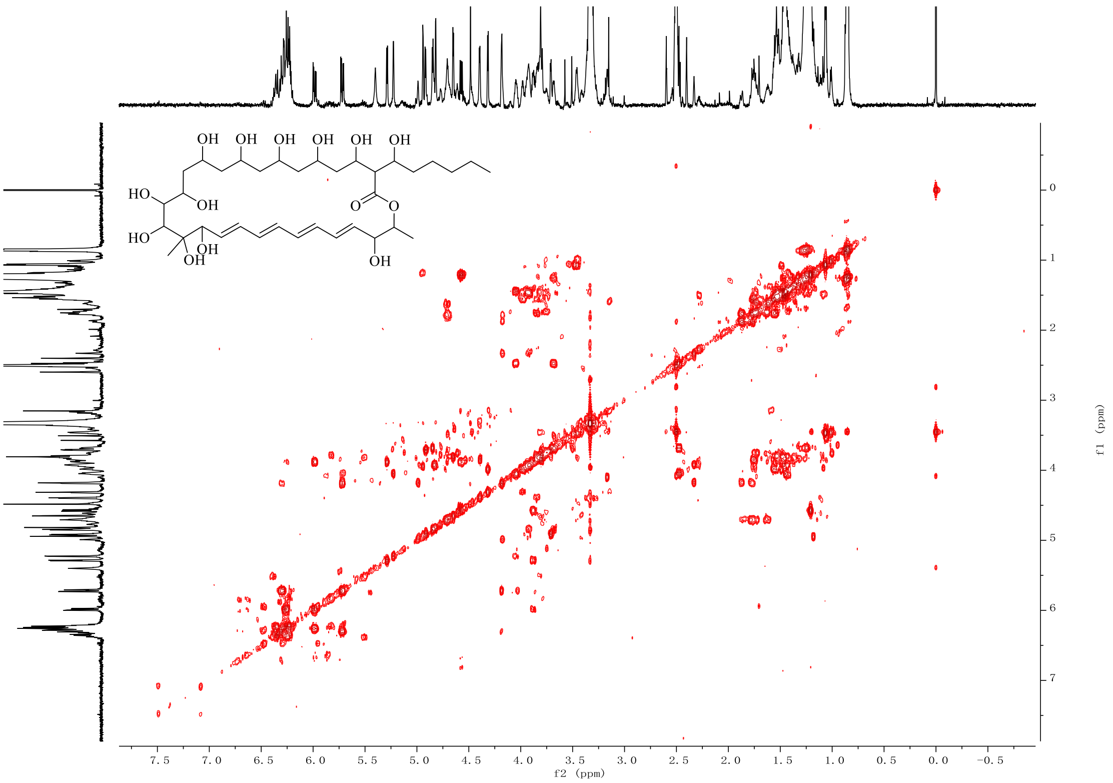

Figure S23. COSY spectrum of **3**.

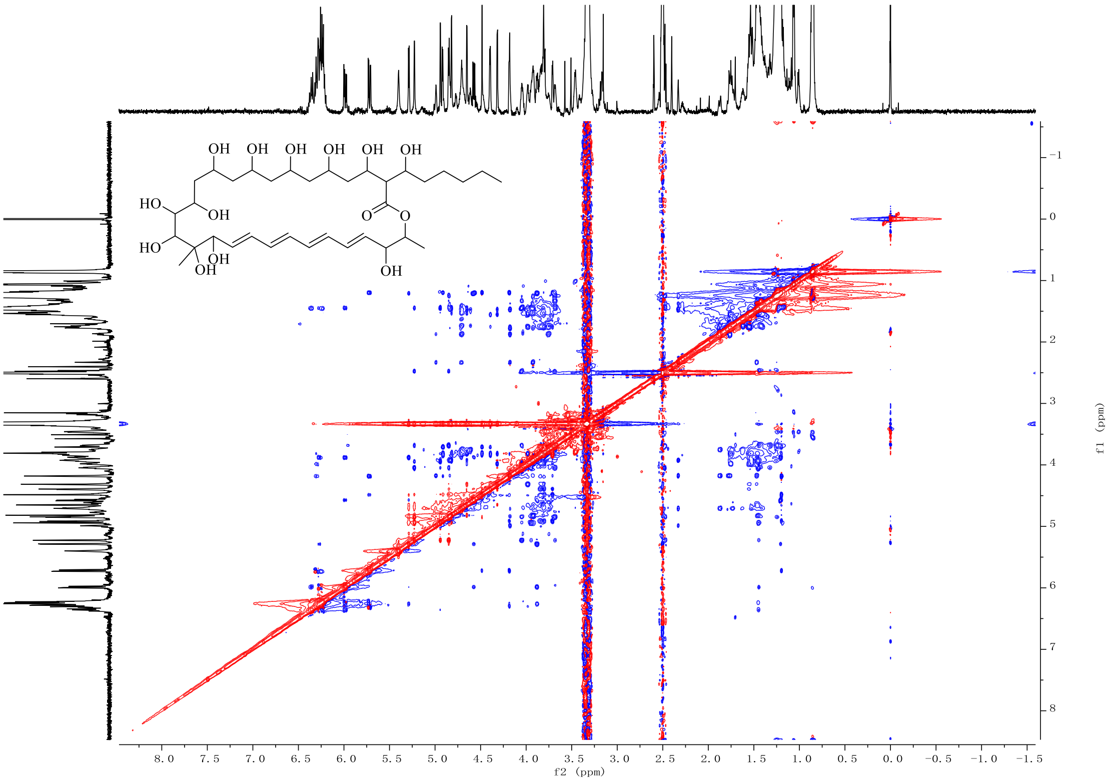

Figure S24. NOESY spectrum of **3**.

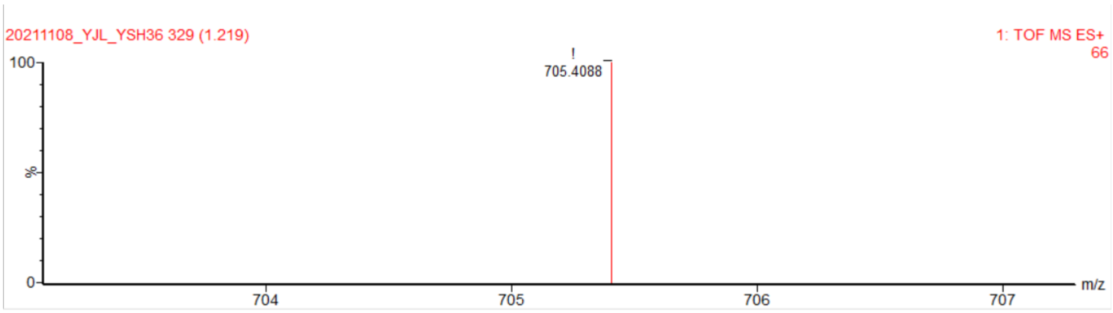

Figure S25. HR-ESI-MS spectrum of **3**.

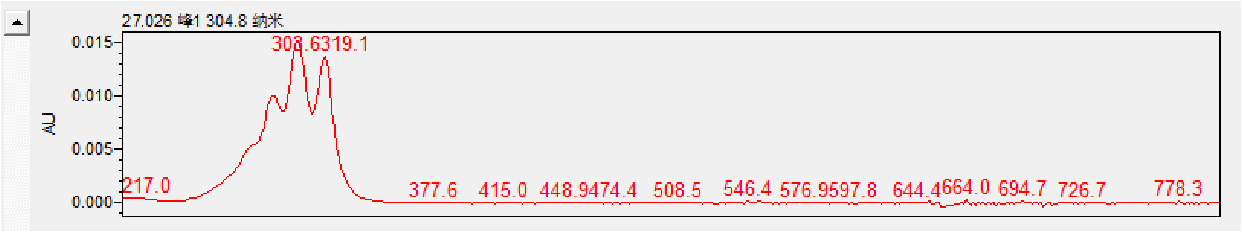

Figure S26. E3xperimental UV spectrum of **3**.

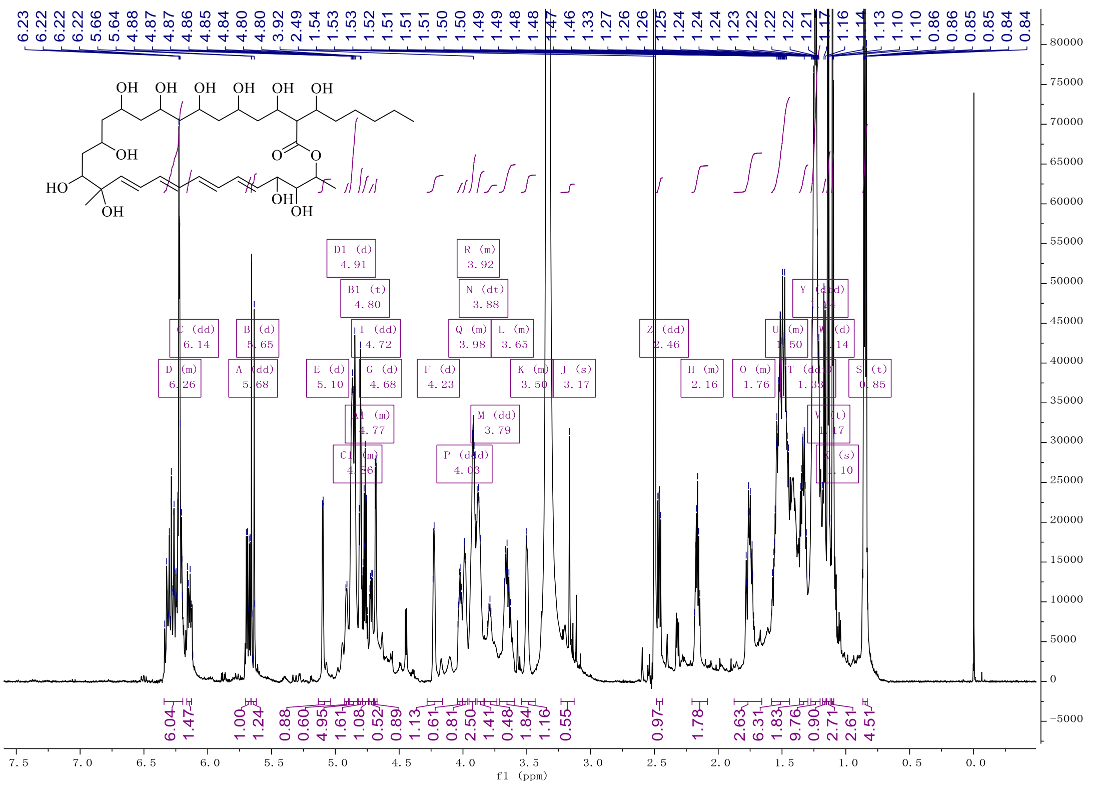

Figure S27. ^{1}H NMR (DMSO-d_6, 700 MHz) spectrum of **4**.

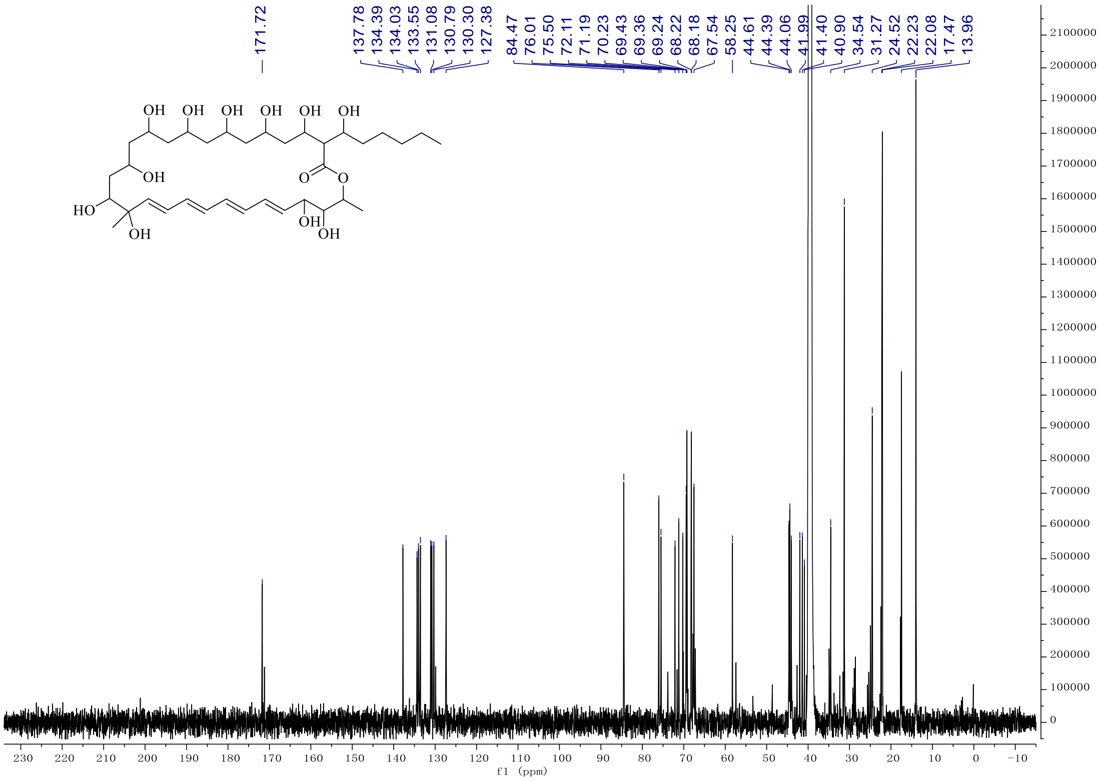

Figure S28. ^{13}C NMR (DMSO-d_6, 175 MHz) spectrum of **4**.

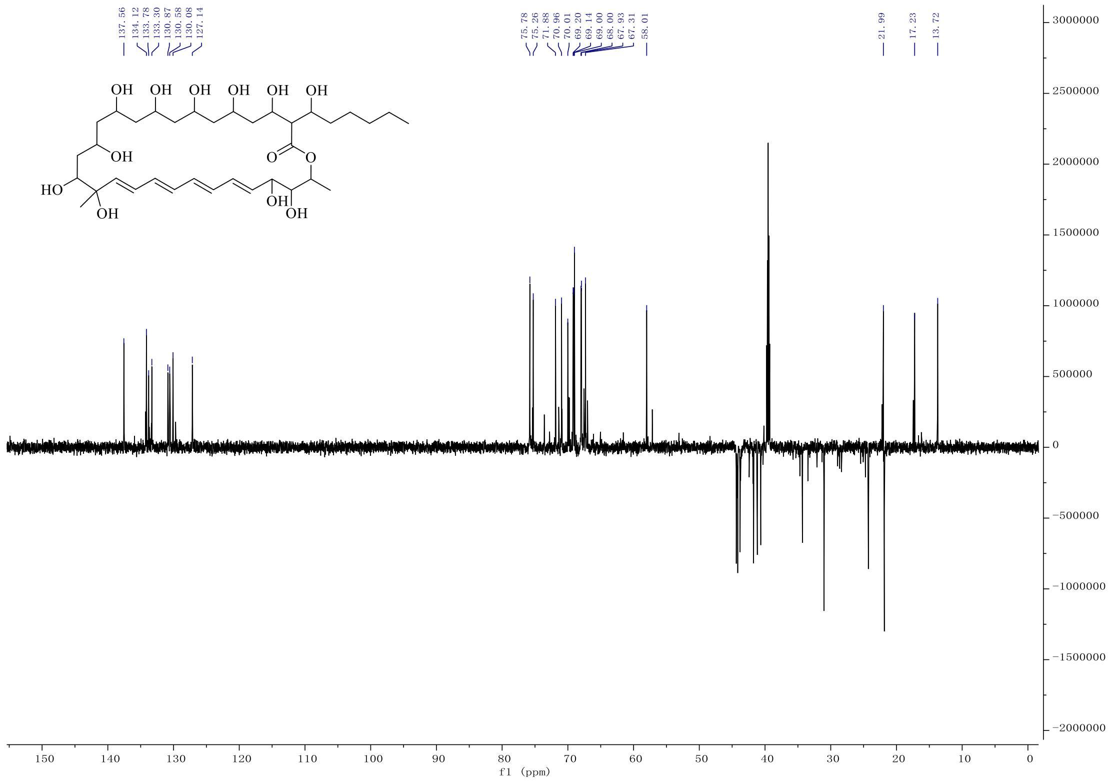

Figure S29. DEPT (DMSO-d_6, 175 MHz) spectrum of **4**.

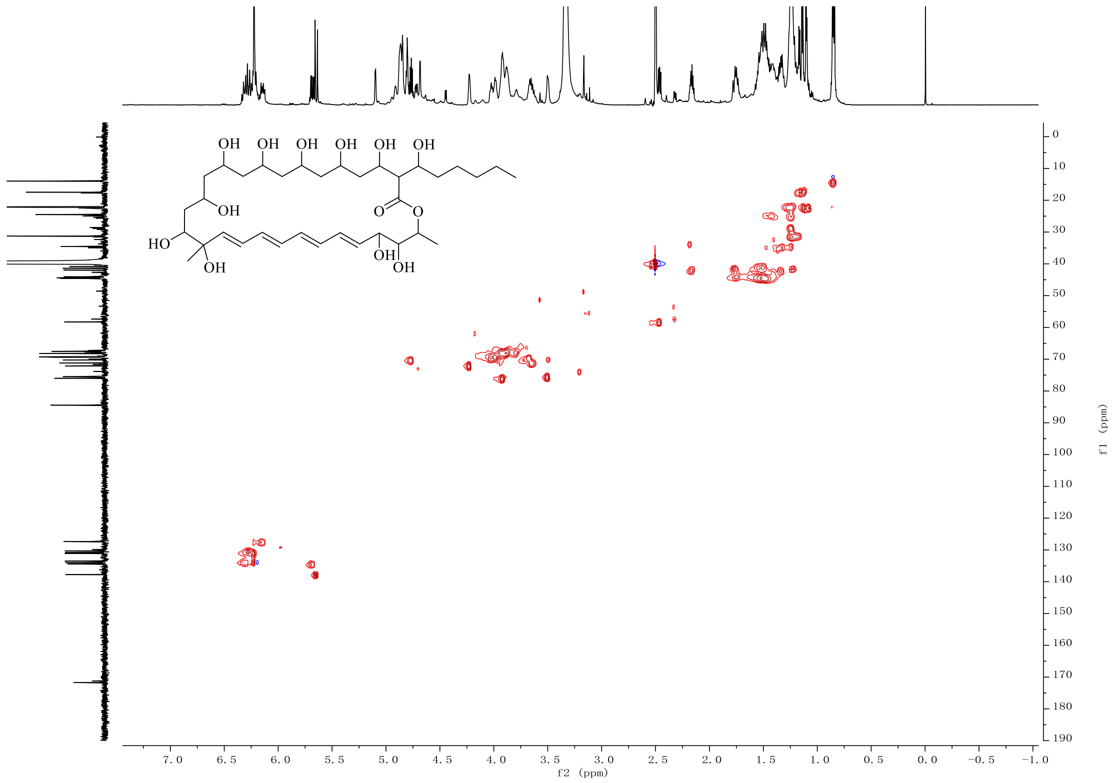

Figure S30. HMQC spectrum of **4**.

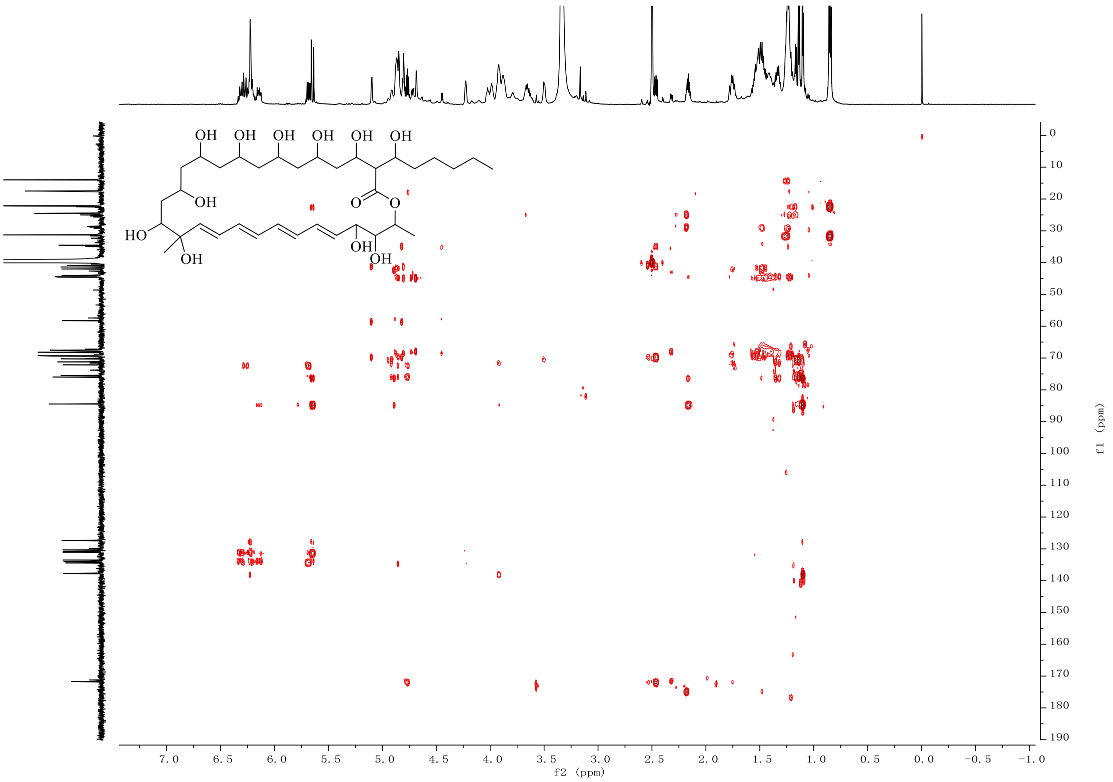

Figure S31. HMBC spectrum of **4**.

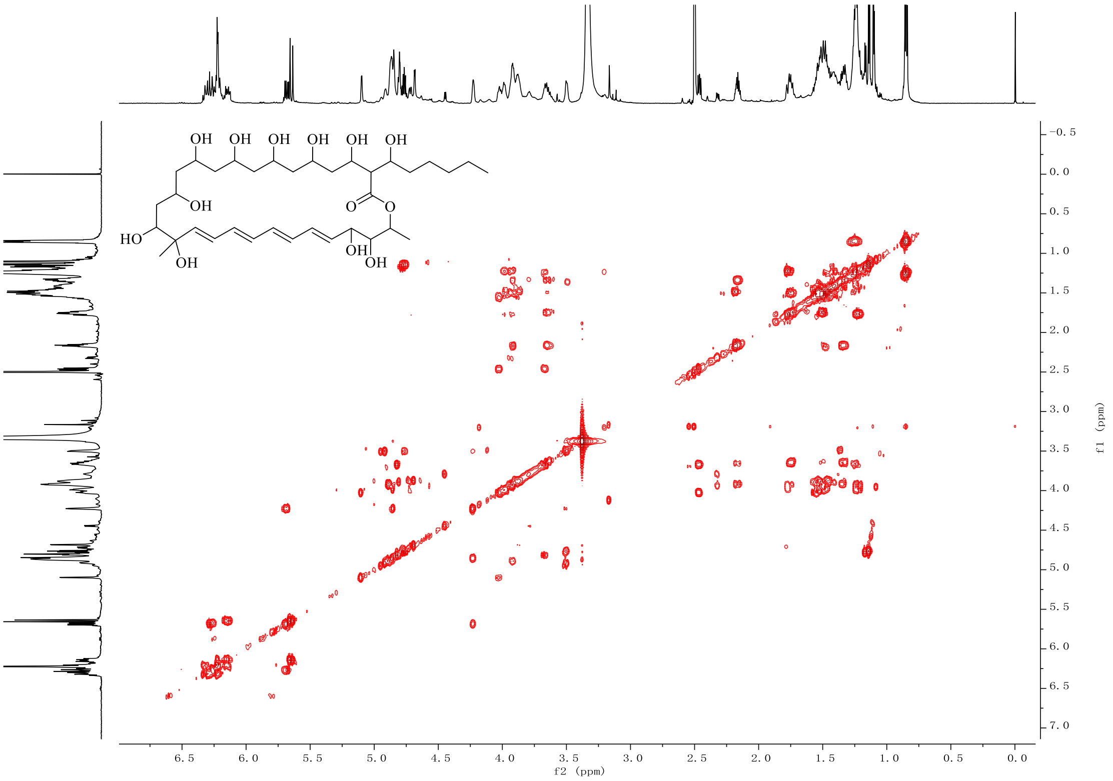

Figure S32. COSY spectrum of **4**.

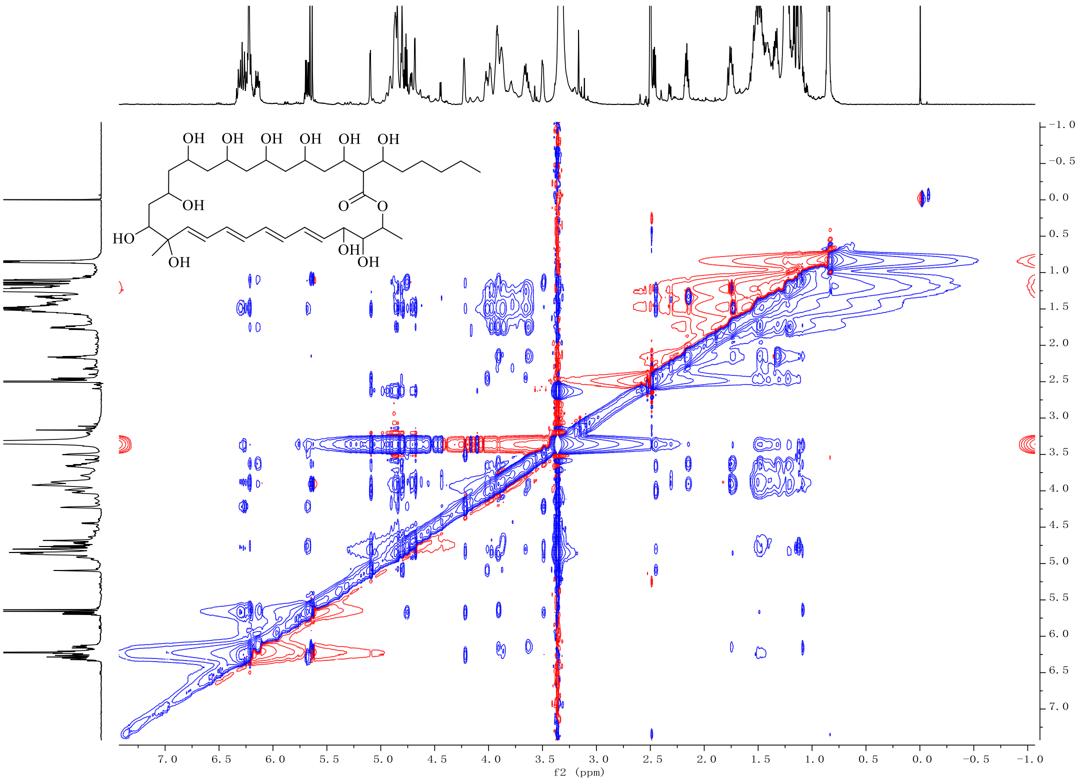

Figure S33. NOESY spectrum of **4**.

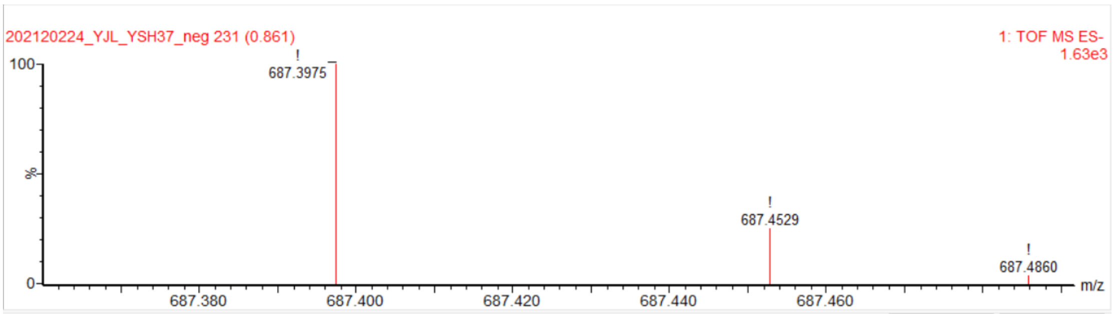

Figure S34. HR-ESI-MS spectrum of **4**.

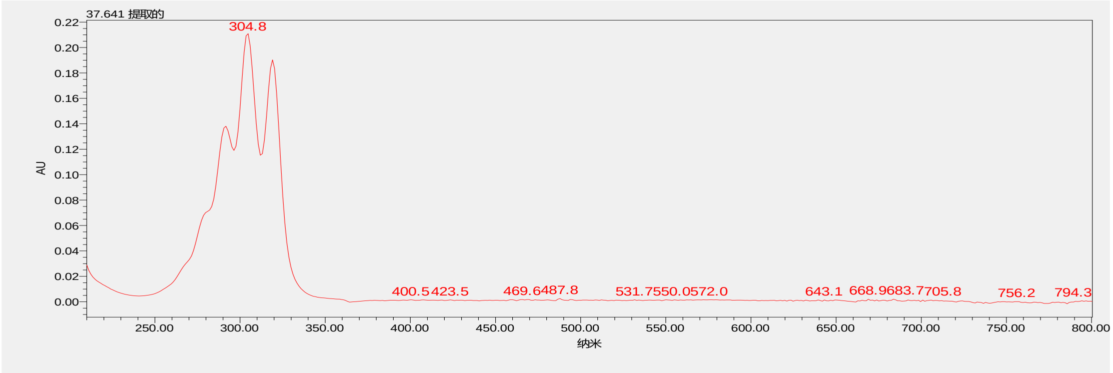

Figure S35. Experimental UV spectrum of **4**.
